# iPromoter-5mC: A Novel Fusion Decision Predictor for the Identification of 5-Methylcytosine Sites in Genome-Wide DNA Promoters

**DOI:** 10.3389/fcell.2020.00614

**Published:** 2020-07-28

**Authors:** Lei Zhang, Xuan Xiao, Zhao-Chun Xu

**Affiliations:** Computer Department, Jing-De-Zhen Ceramic Institute, Jingdezhen, China

**Keywords:** promoter, 5-methylcytosine, fusion decision, predictor, web-server, deep neural network

## Abstract

The hypomethylation of the whole cancer genome and the hypermethylation of the promoter of specific tumor suppressor genes are the important reasons for the rapid proliferation of cancer cells. Therefore, obtaining the distribution of 5-methylcytosine (5mC) in promoters is a key step to further understand the relationship between promoter methylation and mRNA gene expression regulation. Large-scale detection of DNA 5mC through wet experiments is still time-consuming and laborious. Therefore, it is urgent to design a method for identifying the 5mC site of genome-wide DNA promoters. Based on promoter methylation data of the small cell lung cancer (SCLC) from the database named cancer cell line Encyclopedia (CCLE), we built a fusion decision predictor called iPromoter-5mC for identifying methylation modification sites in promoters using deep neural network (DNN). One-Hot Encoding (One-hot) was used to encode the promoter samples for the classification. The method achieves average AUC of 0.957 on the independent testing dataset, indicating that our predictor is robust and reliable. A user-friendly web-server called iPromoter-5mC could be freely accessible at http://www.jci-bioinfo.cn/iPromoter-5mC, which will provide simple and effective means for users to study promoter 5mC modification. The source code of the proposed methods is freely available for academic research at https://github.com/zlwuxi/iPromoter-5mC.

## Introduction

DNA methylation dominates any cell processes, and plays a particularly important role in regulating expression of gene (Bird, 2007; Deichmann, 2016; Nicoglou and Merlin, 2017). DNA methylation at promoters and enhancers has been associated with cell differentiation, developmental processes, cancer development, and regulation of the immune system (Muller et al., 2019). At present, N6-methyladenine (6mA), N4-methylcytosine (4mC) and 5-methylcytosine (5mC) are the three most well-studied types of DNA methylation (Wei et al., 2019). 5mC is a covalent addition between the methyl group and the 5-carbon of the cytosine ring. In somatic cells, 5mC occurs almost exclusively in the context of paired symmetrical methylation of a CpG site.

Recent study (Michalak et al., 2019) suggests that aberrant levels of 5mC at CpG islands in promoter regions is associated with inactivation of various tumor suppressor genes (TSGs). In young normal cells, 5mC is low in the promoter regions but high in the genic and intergenic regions. However, in aging and in cancer, a limited number of genomic loci acquire 5mC, especially at the CpG islands in promoter regions of tumor suppressor and Polycomb-repressed gene, resulting in gene silencing and loss of function. In normal tissue, heterochromatin contains repeating elements and is highly methylated. The aberrant promoter methylation can lead to cancer initiation and progression, which has been confirmed in CpG island methylator phenotype (CIMP) cancers (Gessler, 1999; Kang et al., 2002; Mansour, 2014). Thus, promoter methylation can be used as a potential biomarker for cancer diagnosis and for helping determine prognosis, indicating that identification of 5mC modification in promoter regions by analyzing CpG islands in cell systems of a specific cancer could provide a reference for cancer early diagnosis and precise treatment.

Among cancers worldwide, both the incidence and death rate of lung cancer are in the first place. Small cell lung cancer (SCLC) poses approximately 15% of newly increasing clinical cases with lung cancer each year (Siegel et al., 2018). Its pattern is significantly different from other lung cancer, and is closely related to the high expression of E2F target and EZH2 gene of histone methyltransferase. Furthermore, SCLC is famous for its dense cluster of high-level methylation in CpG islands of discrete promoter. Therefore, in this study, we are concentrating on improving the ability to access the methylation status of promoters for a large number of genes or the entire genome in SCLC.

One of the most usual methods for identifying DNA methylation is distinguishing the cytosine-5 methylation within the CpG dinucleotides (Bianchi and Zangi, 2015; Muller et al., 2019). The popular sequencing technology for identifying 5mC sites includes Methylated DNA immunoprecipitation sequencing (MeDIP-seq), Methyl-Binding Domain sequencing (MBD-seq) and DNA methylome profiling at single-base resolution through bisulfite sequencing (MB-seq) (Down et al., 2008). However, these wet-lab methods are expensive and time-consuming. Therefore, it is urgent to develop a number of methods or tools for the accurate detection of DNA 5mC modification sites.

Over the past decade, computational methods have been proposed to identify 5mC modification sites. Bhasin et al. (2005) developed a SVM-based model called “Methylator,” for the prediction of 5mC modification sites using the methylated and non-methylated CpG dinucleotide sequences from various sources ranging from plants to humans in MethDB database (Amoreira, 2003). Fang et al. (2006) developed a SVM-based classifier called “MethCGI” using nucleotide sequence contents and transcription factor binding sites as features. Compared with the previous two, the predictor “iDNA-Methyl” (Liu et al., 2015) constructed by using the trinucleotide composition and pseudo amino acid components achieved higher success rates. Recently, a novel computational tool called NanoMod (Liu et al., 2018) was designed to improve the performance of detecting candidate positions with DNA modifications. Based on deep neural networks, a computational approach called DeepCpG (Angermueller et al., 2017) was developed to predict methylation states in single cells.

Though the research about the recognition of DNA 5mC modification sites have had a significant advance in recent years, but still exist shortness. Compared to increasing massive high-throughput data, previous studies are of small sample size. Furthermore, among above-mentioned methods, there are three webservers developed by the researchers: Methylator, MethCGI, and iDNA-Methyl, however, only the latter is available but slow, causing much inconvenience to scholars. Most importantly, there is still no computation tool to identify DNA 5mC modification sites in promoters to detect the biomarkers of a specific cancer. Therefore, in the current study, we are devoted to solve these problems and to develop a tool or software for quickly and precisely identifying DNA 5mC modification sites in promoters.

## Materials and Methods

### Benchmark Datasets

The construction of the high-quality data sets is an essential step in the process of establishing the classification model. In the current study, all the sequence samples were collected from the database named cancer cell line Encyclopedia (CCLE) (Barretina et al., 2012; Li et al., 2019), which provided the location information of gene promoter regions and 5mC modification sites experimented by reduced representation bisulfite sequencing (RRBS) (Ghandi et al., 2019) in cell lines of various cancers. Due to the high incidence rate and mortality rate of lung cancer, here we focused on the small cell lung cancer (SCLC) to reveal the distribution of 5mC modification in promoters.

In accordance with the forward/reverse (±) chain and 5mC modification sites' positions in promoters, we collected the sequence samples from the most recent human assembly GRCh37/hg19 on UCSC Genome Browser. It is noteworthy that the sample sequence containing 5mC modification site described as the base G (guanine) in the reverse chain should convert to the reverse complementary sequence, compatible with the principle that the DNA 5mC methylation tends to occur at cytosine (C). Generally, we considered the base C with the methylation level greater than zero as the true 5mC modification site, otherwise, as the false 5mC modification site.

In order to more succinctly describe the promoter sequence fragment potentially containing 5mC modification site, the sample sequence can be expressed as

(1) $$\begin{array}{l} E_{\delta }\left(\mathbb{C}\right)=E_{-\delta }E_{-\left(\delta -1\right)}\cdot \cdot \cdot E_{-2}E_{-1}\mathbb{C}E_{+1}E_{+2}\cdot \cdot \cdot E_{+\left(\delta -1\right)}E_{+\delta } \end{array}$$

where the double letter ℂ represents the cytosine; the subscript δ is an integer, indicating the location of the base in the sequence; *E*_−δ_ is the δ -th base upstream from the center and *E*_+δ_ is the δ -th base downstream from the center.

The sample sequence thus obtained can be divided into two categories:

(2) $$\begin{array}{l} E_{\delta }\left(\mathbb{C}\right)\in \{\begin{array}{c} E_{\delta }^{-}\left(\mathbb{C}\right)\\ E_{\delta }^{+}\left(\mathbb{C}\right) \end{array} \end{array}$$

where $E_{\delta }^{-}\left(\mathbb{C}\right)$ represents a false 5mC modification segment with ℂ at its center, $E_{\delta }^{+}\left(\mathbb{C}\right)$ denotes a true 5mC modification segment with ℂ at its center, and the symbol ∈ denotes “a member of” in the set theory.

Therefore, the benchmark dataset can be formulated by

(3) $$\begin{array}{l} S_{\delta }=S_{\delta }^{-}\cup S_{\delta }^{+} \end{array}$$

where $S_{\delta }^{-}$ denotes the negative subset containing the false 5mC modification site samples; $S_{\delta }^{+}$, the positive subset containing the true 5mC modification site samples; and symbol ∪ represents union in the set theory.

Unbalanced data between the true 5mC modification site samples and the false 5mC modification site samples could more objectively reflect the distribution of 5mC modification in promoters. Therefore, the proportion of positive samples and negative samples was set to about 1:11 in this study. In order to reduce the adverse effects of redundancy and homologous bias, sequences with more than 80% sequence similarity were removed using CD-HIT software.

Finally, we obtained the benchmark dataset *S*_δ_ composed of 893,326 methylation sample sequences in promoter regions, of which 69,750 sample sequences belong to the positive sample dataset $S_{\delta }^{+}$ and 823,576 sample sequences belong to the negative sample dataset $S_{\delta }^{-}$. To investigate the stability and robustness of the prediction model, we randomly selected 80% data in $S_{\delta }^{+}$ and $S_{\delta }^{-}$, respectively, as training set S_1_ for constructing and training the prediction model, and remained 20% as independent testing dataset S_2_ to test the constructed model (Table 1). These datasets can be downloaded from the website http://www.jci-bioinfo.cn/iPromoter-5mC/download.

**Table 1:** Distribution of experimental data sets.

**Attribute**	**Total**	**Training data**	**Testing data**
Positive	69,750	55,800	13,950
Negative	823,576	658,861	164,715

### Extract Features From DNA Sequences

Feature extraction, fusion and selection are the important steps in machine learning process. Many feature extraction methods for protein, RNA and DNA sequences, including PseAAC, PseKNC, PCPs, PCM, PS(k-mer)NP (Zou et al., 2016), have been proposed to overcome the prediction problem of modification sites. In the current study, we employed two effective feature extraction methods (one-hot and DPF) to extract feature directly form DNA sample sequences.

#### One-Hot Encoding Method (One-Hot)

**Figure 1:**
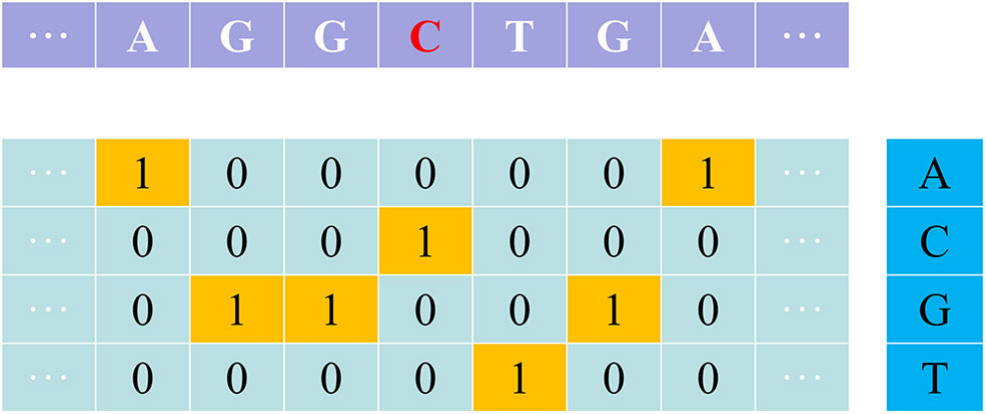
An illustration showing the one-hot encoding method.

One-hot is a simple but effective feature extraction method, especially for deep learning model. The nucleotides A, C, G and T are denoted as one of the four one-hot vectors [1,0,0,0], [0,1,0,0], [0,0,1,0], and [0,0,0,1] (Figure 1).

#### The Deoxynucleotide Property and Frequency (DPF)

Deoxynucleotides are the basic structural and functional units of DNA, and the sequence generated by deoxynucleotides determines biological diversity. Therefore, their chemical properties can influence the inherited characteristics of the DNA sequence to a certain extent. Similar to the encoding method of RNA sequences used in identifying 4mC sites, the deoxynucleotide property and frequency (DPF) (Xia et al., 2019; Xu et al., 2019) is an effective sequence encoding scheme for computationally identifying 5mC modification sites.

Each of the four deoxynucleotides has a different chemical property. Given the sample sequence Q represented by Equation (1), the *k*-th deoxynucleotide in Equation (1) can be converted into a three-dimensional vector, as shown in the Equation (4). Considering that purines have two rings between them and pyrimidines have only one ring, we added the feature of ring structure to feature extraction. Since there is an amino group between A and C, but A keto group between G and T, we added functional group features to feature extraction. In terms of the strength of the hydrogen bond between the base pair, the hydrogen bond between C and G is stronger than the hydrogen bond between A and T, because A is always paired with T by two hydrogen bonds, but C is bound to G by three hydrogen bonds. So we added hydrogen bond features to Q, as shown in the following expression.

(4) $$\begin{array}{l} Q_{k}=\left(x_{k},y_{k},z_{k}\right) \end{array}$$

where *x*_*k*_ represents the “ring structure”; *y*_*k*_, the “functional group”; *z*_*k*_, the “hydrogen bond.”

*x*_*k*_, *y*_*k*_ and *z*_*k*_ can be formulated by Equation (5):

(5) $$\begin{array}{l} x_{k}=\{\begin{array}{c} 1\;if\;Q_{k}\in \left\{A,G\right\}\\ 0\;if\;Q_{k}\in \left\{C,T\right\} \end{array}\\ y_{k}=\{\begin{array}{c} 1\;if\;Q_{k}\in \left\{A,C\right\}\\ 0\;if\;Q_{k}\in \left\{G,T\right\} \end{array}\\ z_{k}=\{\begin{array}{c} 1\;if\;Q_{k}\in \left\{A,T\right\}\\ 0\;if\;Q_{k}\in \left\{C,G\right\} \end{array} \end{array}$$

In order to extract the sequence position information as much as possible (Chen et al., 2017), the cumulative frequency characteristics of deoxynucleotides were adopted:

(6) $$\begin{array}{l} \lambda_{k}=\frac{\sum_{j=1}^{k}F\left(M_{j}\right)}{k}\;\left(1\leq k\leq 2\delta +1\right) \end{array}$$

where *k* is the length of the sample sequence, λ_*k*_ is the density of the deoxynucleotide *Q*_*k*_ along the subsequence from position 1 to position *k* in the sample sequence, and $F\left(M_{j}\right)$ can be expressed as below.

(7) $$\begin{array}{l} F\left(M_{j}\right)=\{\begin{array}{c} 1\;if\;M_{j}=Q_{k}\\ 0\;otherwise\; \end{array} \end{array}$$

Then we obtained a feature vector $\vec{\nu }$ to represent the *k*-th deoxynucleotide in the sample sequence, as shown in the following formula,

(8) $$\begin{array}{l} \vec{\nu }=\left(x_{k},y_{k},z_{k},\lambda_{k}\right) \end{array}$$

The chemical properties of deoxynucleotides reveal the intrinsic relationship between the four different deoxy nucleotides in the sequence and represent the sequence information as discrete vectors by means of 0–1 coding. Therefore, by this method, we represented the sequence with 4 × *L*-D (dimensional) feature vector W to represent the sample sequence formulated by Equation (1),

(9) $$\begin{array}{l} W={\left[x_{1}y_{1}z_{1}\lambda_{1}\cdot \cdot \cdot \;x_{2\delta +1}\;y_{2\delta +1}\;z_{2\delta +1}\;\lambda_{2\delta +1}\right]}^{T} \end{array}$$

where the symbol T is the transpose operator.

### Feature Fusion

Feature fusion usually joins several kinds of different feature vectors into an integrated one, which could express the local and global sequence order information of the given sample sequence. Therefore, in this study, we not only employed one-hot and DPF methods, but also took into account their combination. According with this method, we represented the sequence with 2 × 4 × *L*-D (dimensional) feature vector.

### Framework of the Integrated Predictor

**Figure 2:**
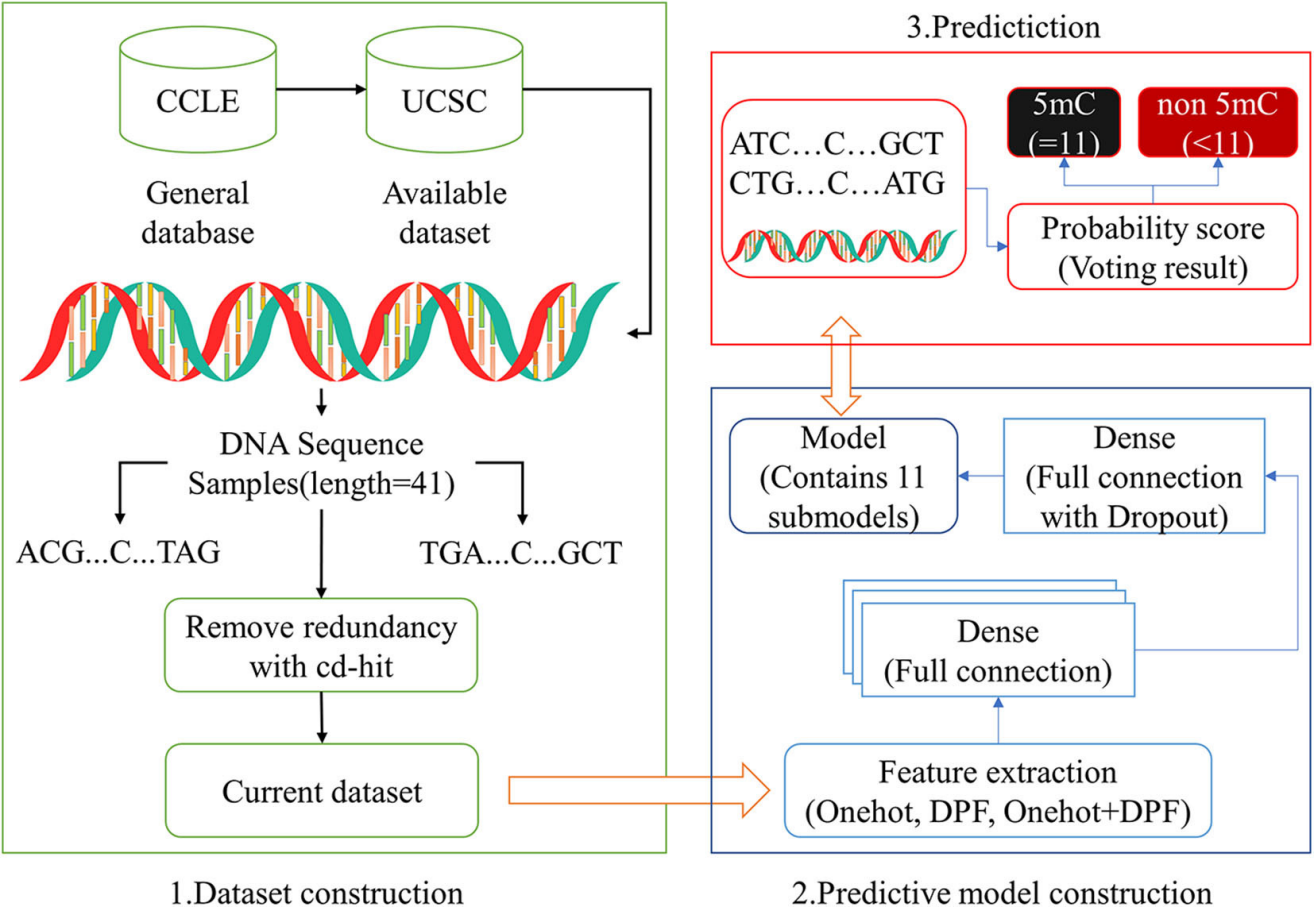
Framework of the integrated predictor.

For imbalance problems existing in positive samples and negative samples, the down-sampling method was adopted in the current study. We randomly divided the negative samples from the training dataset S_1_ into 11 groups of equal size, one of which can form the balance training subset by combining with the positive samples in the same amount. And then, we could obtain 11 sub-models. After converting into a numeric vector by one-hot, DPF or their combination, a query sequence with the base C in its center, can be input into 11 sub-models for prediction. The 11 prediction results thus obtained can be used to generate the final decision whether the base C is methylated or not by some judging methods, just like a simple majority vote or weighted voting method (Figure 2). The integrated predictor obtained by above-mentioned method was named as iPromoter-5mC, which can be used to identify the 5mC modification sites in promoter sequences.

In this study, a simple deep neural network (DNN) framework (Islam et al., 2018) was employed to consturcted the prediction model. The generated feature matrix was fed into the fully connected neural network for training. The fully connected layer of DNN contained 64, 128, 256, 128, 64 neurons in turn, and the activation function was ReLU (Zhuang et al., 2019). For binary problem, the last layer contained two neurons, and sigmoid was selected as the activation function. To prevent overfitting and improve model generalization, a dropout layer was added before the last full connection layer, with a value of 0.3. Five-fold cross validation was conducted to validate the reliability of each sub-model.

### Evaluation Metrics

K-fold cross-validation method could effectively utilize limited data, and the evaluation results are as close as possible to the model's performance on the test set. Therefore, we used this method to evaluate the model's performance (Wei et al., 2018; Chen et al., 2019a,b; Dao et al., 2019). For single label system, there are several common evaluation indexes to measure the predictive performance of the predictor, including Sensitivity (Sn), Accuracy (Acc), Specificity (Sp) and Matthew's correlation coefficient (MCC), which can be defined as following,

(10) $$\begin{array}{l} \{\begin{array}{ccc} Sn & = & 1-\frac{N_{-}^{+}}{N_{+}},0\leq Sn\leq 1\\ Sp & = & 1-\frac{N_{+}}{N},0\leq Sp\leq 1\\ Acc & = & 1-\frac{N_{-}^{+}+N_{+}}{N^{+}+N},0\leq Acc\leq 1\\ MCC & = & \frac{1-\left(\frac{N_{-}^{+}+N_{+}}{N^{+}+N}\right)}{\sqrt{\left(1+\frac{N_{+}N^{+}}{N^{+}}\right)\left(1+\frac{N_{-}^{+}N_{+}}{N}\right)}},0\leq MCC\leq 1 \end{array} \end{array}$$

where *N*^+^ is the total number of 5mC sites actually containing in the sample sequences, i.e., the sum of the quantities of true positive; while *N*^−^ denotes the total number of non-5mC site sequences, i.e., the sum of the quantities of true negative; $N_{-}^{+}$ represents the number of true 5mC sites predicted incorrectly as non-5mC sites; $N_{+}^{-}$ represents the number of non-5mC sites predicted incorrectly as true 5mC sites.

In addition, we used the Receiver Operating characteristic curve (ROC curve) to exam the performance of the entire integrated predictor model. The true positive rate (Sn) and false positive rate (1-Sp) were set to x-axis and y-axis to plot the ROC curve, respectively. The area under the ROC curve, also known as AUC, was used to quantify the performance of the model.

## Results and Conclusions

### Window Size Analysis

**Figure 3:**
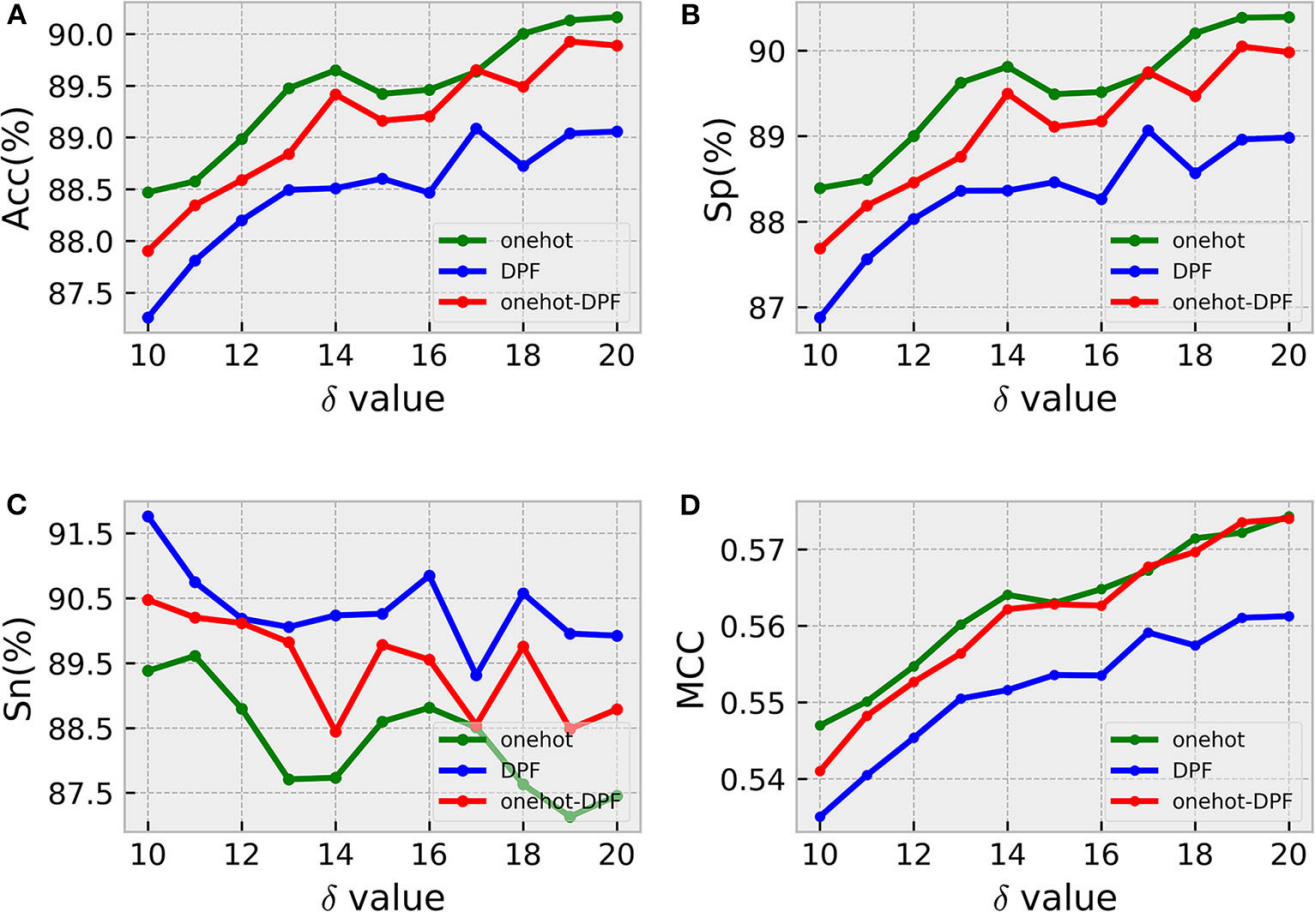
Windows size analysis. Sub-graphs from **(A–D)** represent the ACC, Sp, Sn, MCC values generated by three different feature coding methods under different sliding window sizes, respectively.

Considering the position specific deoxynucleotide bias, it is necessary to determine the optimal window size δ of sample sequences for identifying 5mC modification sites. Generally, if δ is too small, the residues around the 5mC modification sites cannot carry enough information, leading to poor prediction effect (Xu et al., 2019). Thus, we analyzed the trend of the precision rate of the constructed model with different window size δ. As shown in Figure 3, the search step size for δ here was 1nt, with a range of 10–20.

According to the intuitive observation in sub-graphs (A), (B), and (D), when δ = 20, the prediction results generated by the three different methods were the best. In order to distinguish the optimal model obtained by using one-hot, DPF and onehot-DPF, we compared the most important metrics Acc and MCC values, and found that the feature method with the best effect was one-hot, as illustrated in sub-graphs (A) and (D). Therefore, the following analysis and calculation were based on δ with 20, indicating the length of the sample sequence formulated by Equation (1) was 41nt.

### Performance of DNN Models

**Figure 4:**
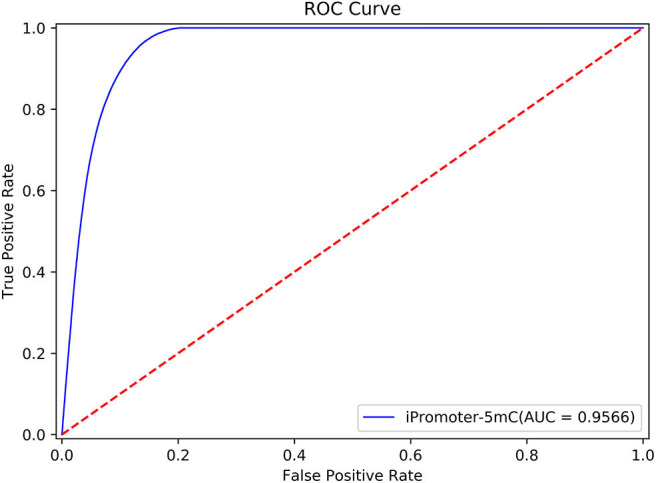
The ROC curve of the S1 dataset on our model.

According to the description in section “Framework of the integrated predictor,” we can construct the 11 sub-models based on the training dataset S_1_ using one-hot feature extraction method. A simple majority vote strategy was used to integrate all the decisions originated from the 11 sub-models into a final classification result. In the current study, we adopted the strict discriminating standard for identifying 5mC modification sites. If only all the sub-models consider that the potential 5mC sites is a true 5mC modification site, the iPromoter-5mC model could infer the center of this query sequence is a 5mC modification site. After 30 repeated experiments with 5-fold cross validation, we obtained the average values of each metric as the final results of the iPromoter-5mC model, as shown in Table 2. The results of the iPromoter-5mC model indicated that the performance of our models was promising, supported by the metric values, such as Sn, 87.46%; Sp, 90.39%; Acc, 90.16%; MCC, 0.5743. To more directly illustrate the performance of the predictor, a ROC curve was plotted using the training dataset S_1_, and its corresponding AUC value was calculated. The high AUC value (0.9566) indicates that our predictor iPromoter-5mC has excellent performance and stable performance in predicting the 5mC site (Figure 4).

**Table 2:** The results obtained by 5-fold cross validation on the training dataset S_1_.

**Method**	**Sn (%)**	**Sp (%)**	**Acc (%)**	**MCC**
iPromoter-5mC	87.46	90.39	90.16	0.5743

**Figure 5:**
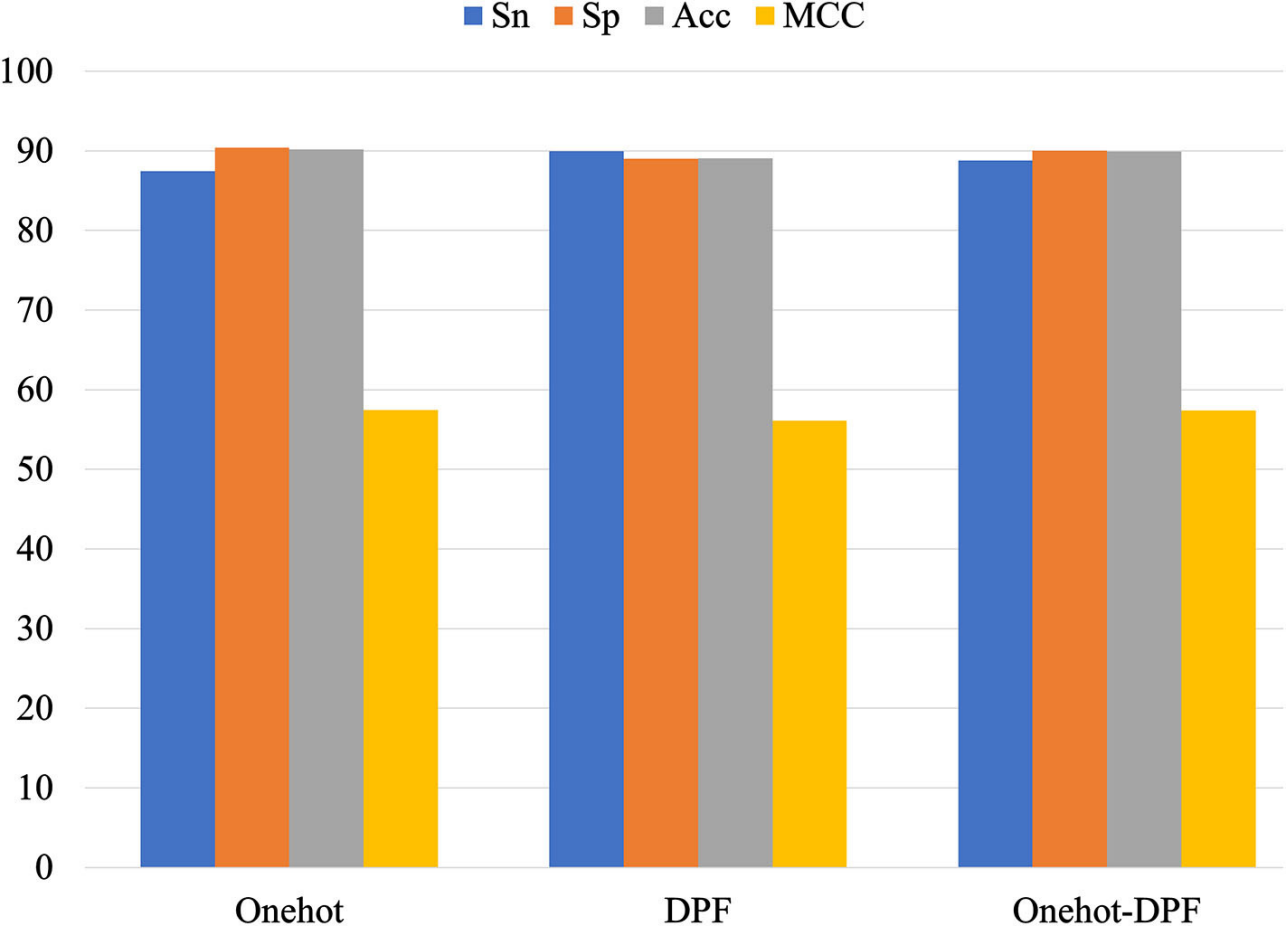
Performance of different feature extraction methods for prediction of 5mC sites.

In order to validate the stability of the DNN algorithm model, we compared the performance of the DNN models constructed by one-hot, DPF, and their combination. All the results were displayed as a histogram directly on Figure 5. Small discrepancies of every metric value obtained by the three different methods indicated the superior stability of the DNN algorithm model to identify the 5mC modification sites.

### The Robustness and Reliability Analysis

Independent test is an effective approach to check the performance of the constructed classification model. Compared with the cross-validation method, it can better verify the robustness and reliability of the prediction models. In the section “Benchmark datasets” in this study, we established the training dataset S_1_ and independent testing dataset S_2_. Here, we used the independent testing dataset S_2_ to further test the performance of the predictor iPromoter-5mC. The results were listed in Table 3.

**Table 3:** The performance of iPromoter-5mC based on the independent dataset.

**Model number**	**Sn (%)**	**Sp (%)**	**Acc (%)**	**MCC**	**AUC**
1	94.48	86.53	87.15	0.5455	0.9543
2	98.32	83.19	84.37	0.5183	0.9542
3	95.88	85.77	86.56	0.5417	0.9545
4	96.97	84.71	85.66	0.5319	0.9533
5	95.49	85.97	86.72	0.5425	0.9539
6	95.59	85.88	86.64	0.5417	0.9542
7	97.84	83.84	84.93	0.5244	0.9531
8	97.94	83.75	84.86	0.5238	0.9535
9	94.24	86.71	87.29	0.5469	0.9539
10	95.98	85.69	86.49	0.5409	0.9542
11	97.53	84.04	85.09	0.5256	0.9545
iPromoter-5mC	87.77	90.42	90.22	0.5771	0.9570

The predictive results of the 11 sub-models using the 5-fold cross-validation method on the independent test dataset S_2_ were very stable at about 95, 83, 85%, 0.52 and 0.95 in Sn, Sp, Acc, MCC, and AUC, respectively, indicating that the constructed sub-models are very robust for identifying 5mC modification sites on new data. After integrating all the decisions originated from these sub-models, the independent test performance of this final model were 87.77, 90.42, 90.22%, 0.5771 and 0.9570 in Sn, Sp, Acc, MCC and AUC, respectively. The performance of the predictor iPromoter-5mC was improved, mainly seen in the metrics Acc and MCC. This implied that our designed framework for 5mC modification site prediction is reasonable and efficient, indicating that this method can be extended to realize synthetic problems on accurate prediction of other DNA/RNA modification sites.

**Figure 6:**
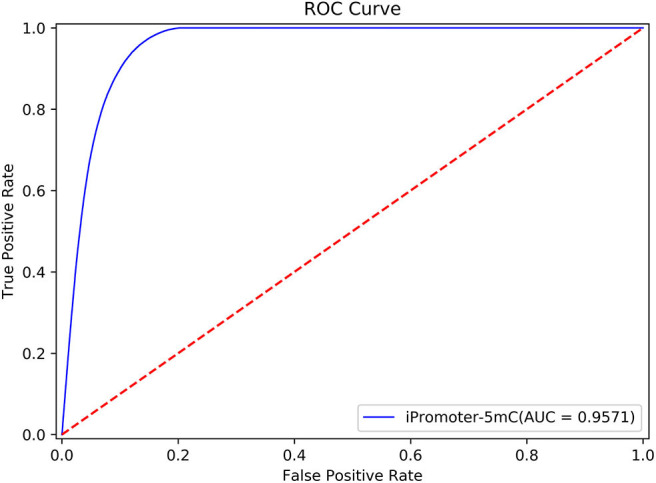
The performance generated by the same prediction framework was still reliable and stable after the expansion of the training data.

To further validate the robustness and reliability of the prediction framework, we implemented 5-fold cross validation on the benchmark dataset *S*_δ_ including the training dataset S_1_ and the independent test dataset S_2_. The results of the ROC curve shown in Figure 6 showed that the performance generated by the same prediction framework was still reliable and stable after the expansion of the training data, which have laid a solid foundation for establishment of online predictor.

We are also concerned with whether our models are applicable to the data from other cell line or tissues. To do so, we firstly constructed the benchmark dataset according to the 5mC site information in promoter regions of human hepatocarcinoma cell lines (HUH7_LIVER) from database CCLE. This dataset also was divided into the training dataset and the independent test dataset, which were also released on the GitHub and on our online server. And then, we constructed the DNN model using the same method proposed in this study. The results listed in Table 4 were also promising, indicating that the method using in this study can also be applied to the prediction of 5mC sites in other cancer cell lines.

**Table 4:** The 5-fold cross validation results on the training set and the independent test set of human hepatocarcinoma cell lines.

**Method**	**Sn (%)**	**Sp (%)**	**Acc (%)**	**MCC**	**AUC**
iPromoter-5mC (training)	80.53	95.79	93.73	0.7408	0.9736
iPromoter-5mC (independent test)	81.22	95.79	93.81	0.7459	0.9735

### Comparison With Existing Predictor

Compared with the two early predictors Methylator and MethCGI, the predictor iDNA-Methyl has better prediction performance, which has been demonstrated in the study (Liu et al., 2015). And iDNA-Methyl has own webserver for identifying DNA 5mC sites. Therefore, we compared the performance of iPromoter-5mC with those of iDNA-Methyl. For convenience of comparison, the scores of the four indexes defined in Equation 10 obtained by these two predictors based on the independent test dataset S_2_ were listed in Table 5. It can be observed from the table that the overall accuracy (Acc) score obtained by the current iPromoter-5mC is significantly higher than that of the existing predictors, as are the other three indicators.

**Table 5:** Comparison of predictors' performance on the independent testing dataset S_2_ and sample data from iDNA-Methyl by 5-fold cross validation, respectively.

**Success rates**	**Dataset S2**		**Sample data from iDNA-Methyl**	
	**iPromoter-5mC**	**iDNA-Methyl**	**iPromoter-5mC**	**iDNA-Methyl**
Sn (%)	87.77	30.62	83.48	61.25
Sp (%)	90.42	90.30	88.04	90.33
Acc (%)	90.22	85.90	86.56	77.49
MCC	0.5771	0.1730	0.7013	0.5471

We analyzed its causes and presently summarized as follows: (1) There is the biggest difference between iDNA-Methl and iPromoter-5mC. From the view of the function, iDNA-Methl detected the genome-wide methylation while iPromoter-5mC identified the methylation sites in promoters. (2) Most importantly, the sizes of their benchmark dataset are significantly different. The sample size of iPromoter-5mC is far greater than iDNA-Methl's, which enables our model to obtain better correlation between sequences, causing the phenomenon that the server iPromoter-5mC can identify the 5mC sites of the benchmark dataset from iDNA-Methl effectively while iDNA-Methl cannot. (3) The other reason is that the non-equilibrium degree of the benchmark datasets from these two predictors is significantly different. The unbalance ratio of the positive samples and negative samples from iDNA-Methyl is about 1:2, however, that of the iPromoter-5mC approximately up to 1:11.

In order to further analyze the performance of these two predictors, we implemented experiments to obtain the result by iPromoter-5mC using the sample data from iDNA-Methyl. And we found that the performance of iPromoter-5mC was better than that of iDNA-Methyl (Table 5), which also benefits from a large amount of data during our training.

In conclusion, these results indicated that deep learning was better suited for identify 5mC sites on a large dataset, compared to SVM. In fact, parameter optimization of SVM is extremely time-consuming, especially in the case of large amount of data. The predictor iPromoter-5mC can be an outstanding supplemental tool for identifying 5mC sites since the predictor iDNA-Methyl.

### Web-Server

A user-friendly web server could provide ease of use for broad scholars to get their desired predictive results without following the complex mathematical calculations. To achieve this, we had developed an online predictor called iPromoter-5mC to identify the 5mC modification sites in promoters, following the principle described below.

For a given promoter sequence, a 41 bp scan window was used to segment the sequence into equal-size sequences. If a DNA query sequence containing potential 5mC modifications sites is in a forward strand, the base C in this DNA sequence will be selected and considered as the fixed length sequence with 41, otherwise, the base G will be found to construct the input sequence, and then be converted to the reverse complementary sequence. After that, users can follow the detailed guide to try out online experience of our web server iPromoter-5mC.

**Figure 7:**
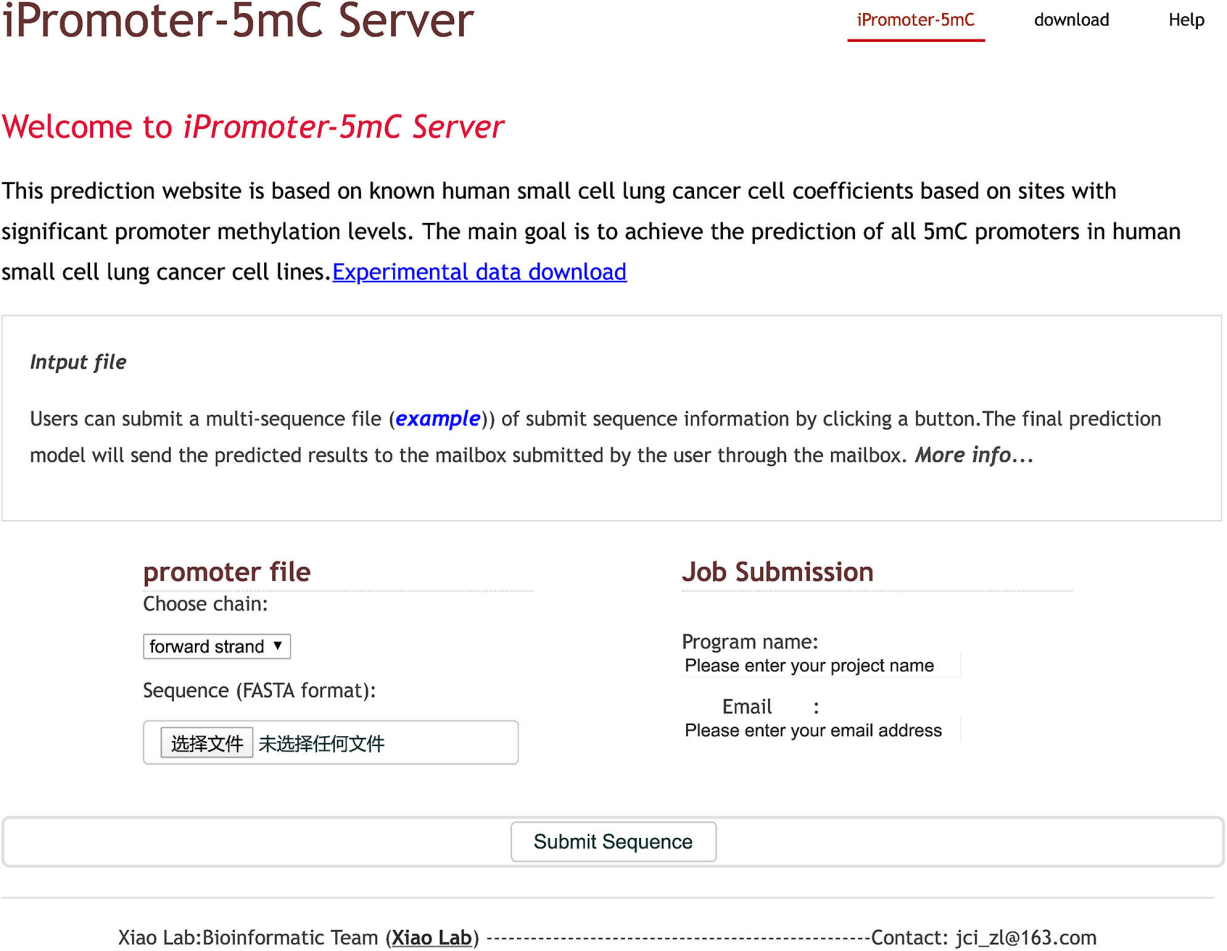
Screen shots of the homepage of the iPromoter-5mC web server.

**Step 1**. Click the link http://www.jci-bioinfo.cn/iPromoter-5mC and then the top page of iPromoter-5mC will be shown in Figure 7.

**Step 2**. Select the strand where the sequence is located from the drop-down list box (the default value is the forward strand).

**Step 3**. Users can submit the file containing multiple sequences in FASTA format by clicking the submit button.

**Step 4**. Enter the project name and your e-mail address. The running results will be sent to you by email after finishing the work.

## Conclusions

In this study, we designed a fast and effective DNN model, named iPromoter-5mC, to identify 5mC modification sites in DNA promoter region in cell lines of the small cell lung cancer. The robustness and good performance of the model were verified by feature analysis and various experiments. More importantly, Due to build an easy to use web server can provide users with more convenient, we set up an online web server to identify 5mC modification sites, which can bring great convenience to scholars' research work. The model mentioned in this paper only targets cell lines of lung small cell carcinoma, but the basic method and analysis flow can also be applied to the prediction of 5mC sites of other cancer cell lines.

Although the model in this study achieved higher predictive performance, the future is going to be one that presents many challenges. We are going to continue to study the predictive problem about DNA 5mC methylation. Firstly, with the development of single cell sequencing technology, we will try to accurately predict single-cell DNA 5mC methylation states using deep learning based on single-cell methylation data. Secondly, we plan to design a scheme to achieve accurate classification of DNA 5mC methylation level. Finally, we will construct machine learning models based on other data in cell lines of other cancers to better detect the biomarkers of those cancers.

## Data Availability Statement

Publicly available datasets were analyzed in this study. This data can be found here: http://www.jci-bioinfo.cn/iPromoter-5mC/download.

## Author Contributions

XX designed the experiments. LZ constructed the predictor and established the online server. Z-CX wrote the manuscript. All authors read and approved the manuscript. In additional, thank Ang Sun for collecting the data information.

## Conflict of Interest

The authors declare that the research was conducted in the absence of any commercial or financial relationships that could be construed as a potential conflict of interest.
